# Supplementary material for: Neural network analysis of sleep stages enables efficient diagnosis of narcolepsy
Source: Nat Commun. 2018 Dec 6;9:5229. doi: 10.1038/s41467-018-07229-3 (PMC6283836; doi:10.1038/s41467-018-07229-3)
Supplement: Supplementary file 1 — Supplementary Information [file 41467_2018_7229_MOESM1_ESM.pdf]

Supplementary Material to Neural network analysis of sleep stages enables efficient diagnosis of narcolepsy

Stephansen and Olesen et al.

SUPPLEMENTARY FIGURES

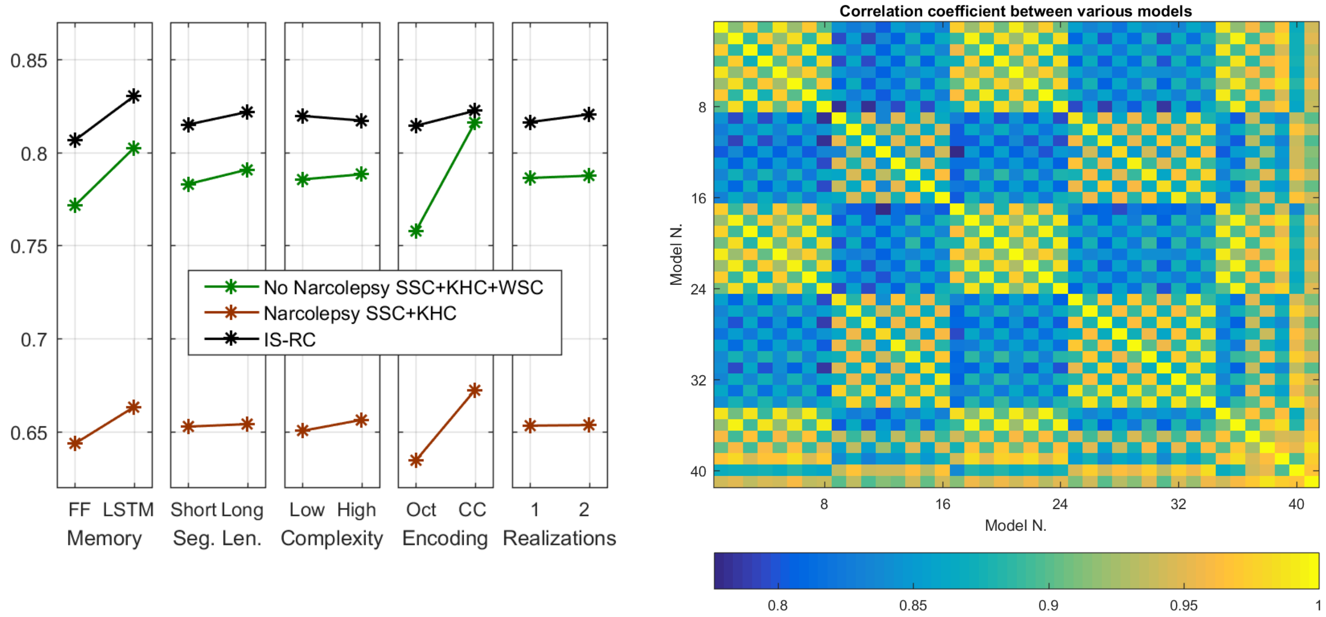

Supplementary Figure 1: Comparisons of machine learning models. Left: Comparisons of the effect on accuracy by each factor at different settings on IS-RC data, SSC and KHC narcolepsy subjects, and the remaining SSC, KHC and WSC subjects used for testing. Right: Correlation matrix showing similarities in different model predictions, where 0 means signals are independent, and 1 means signals are completely correlated. Models number (N) 1-32 are single models, and 33-41 are ensembles. The models vary on 5 parameters, each at two levels, in the following order: Memory – FF or LSTM (1), segment length (Seg. Len.) – 5 s or 15 s (2), complexity – high or low (3), encoding – CC or octave (4), realizations – 1 or 2 (5). Ensembles are as described in Supplementary Table 8: All FF octave models (33), all LSTM octave models (34), all FF CC models (35), all LSTM CC models (36), all FF models (37), all LSTM models (38), all CC models (39), all octave models (40), all models (41).

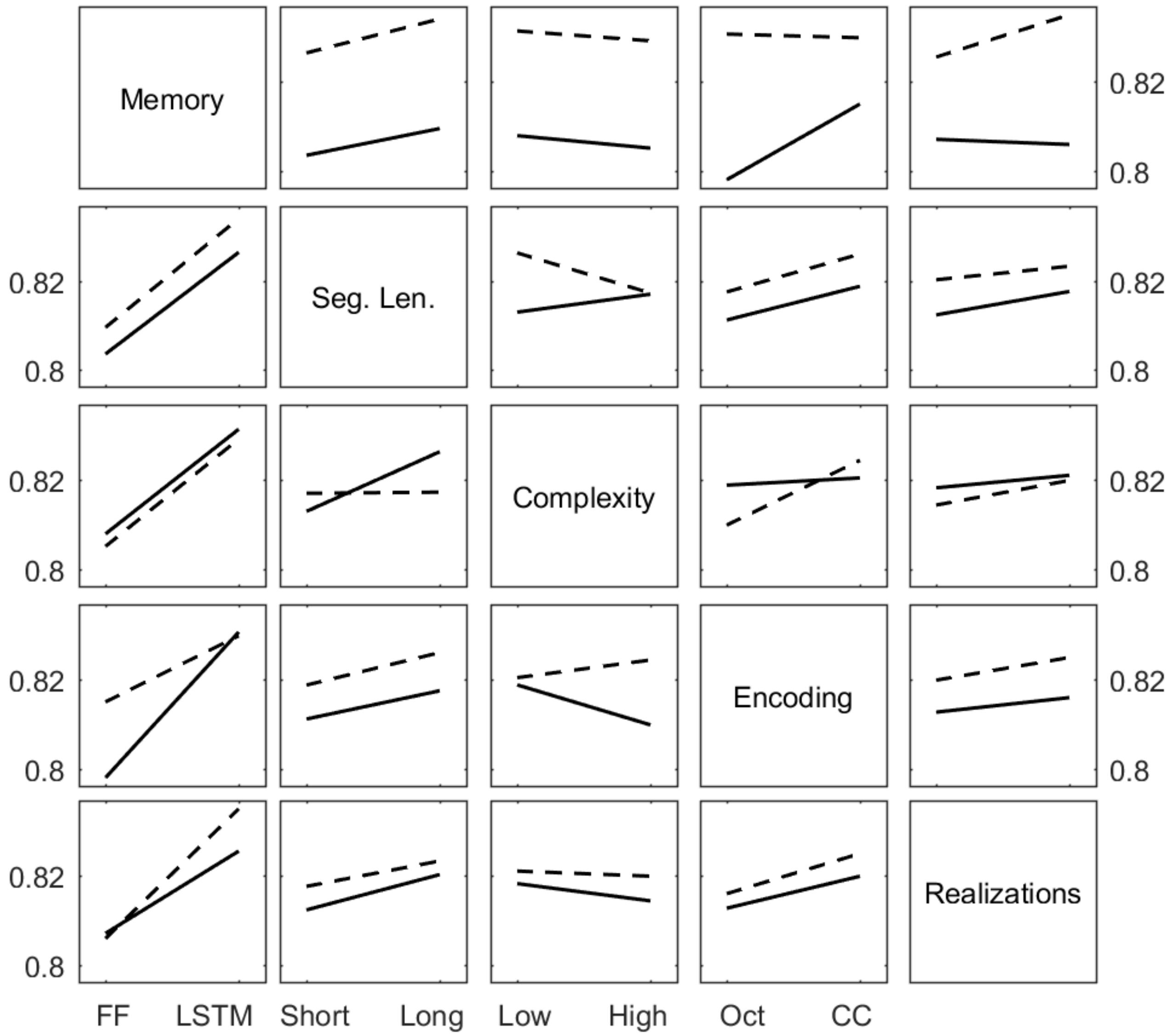

Supplementary Figure 2: Interaction of different factors and their dependence on accuracy. The IS-RC data was used for this analysis. The solid and dashed lines indicate factors along the rows on levels 1 and 2, respectively.

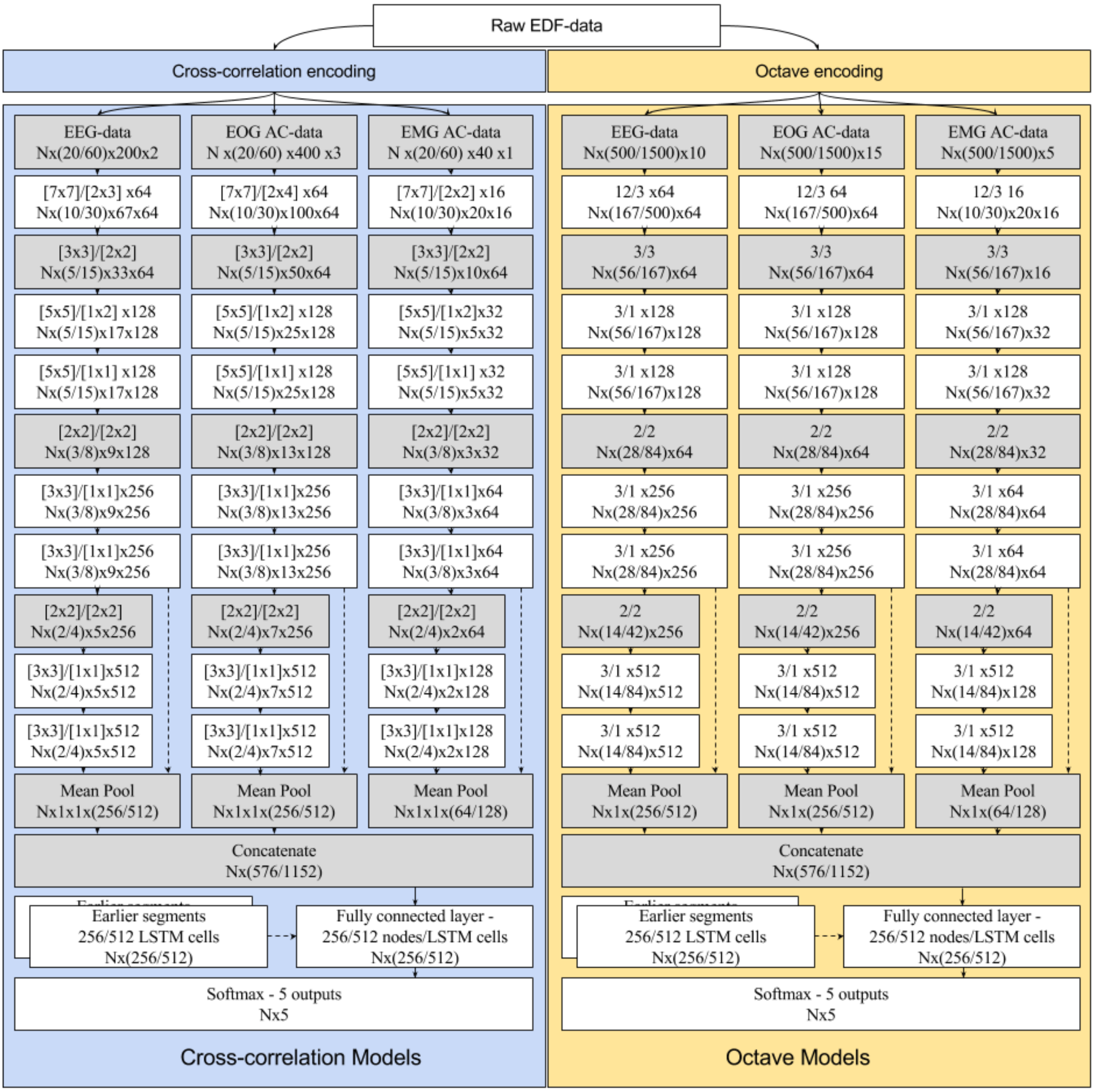

Supplementary Figure 3: Specifications of each network configuration. Each block represents an operation; with white blocks require multiplications and adding, whereas grey blocks are pooling or concatenations, default being max pooling. The top row of each block describes the size of the window and its stride, and the bottom row describes the size of the output. In this output, N is the length of a sequence, the second dimension is the segment length, and if a fourth dimension is present (CC models), the third dimension originally represents the size of the correlation function. The last dimension is the number of features in that layer. Models with a low complexity skip the third max pooling block, and go straight to mean pooling.

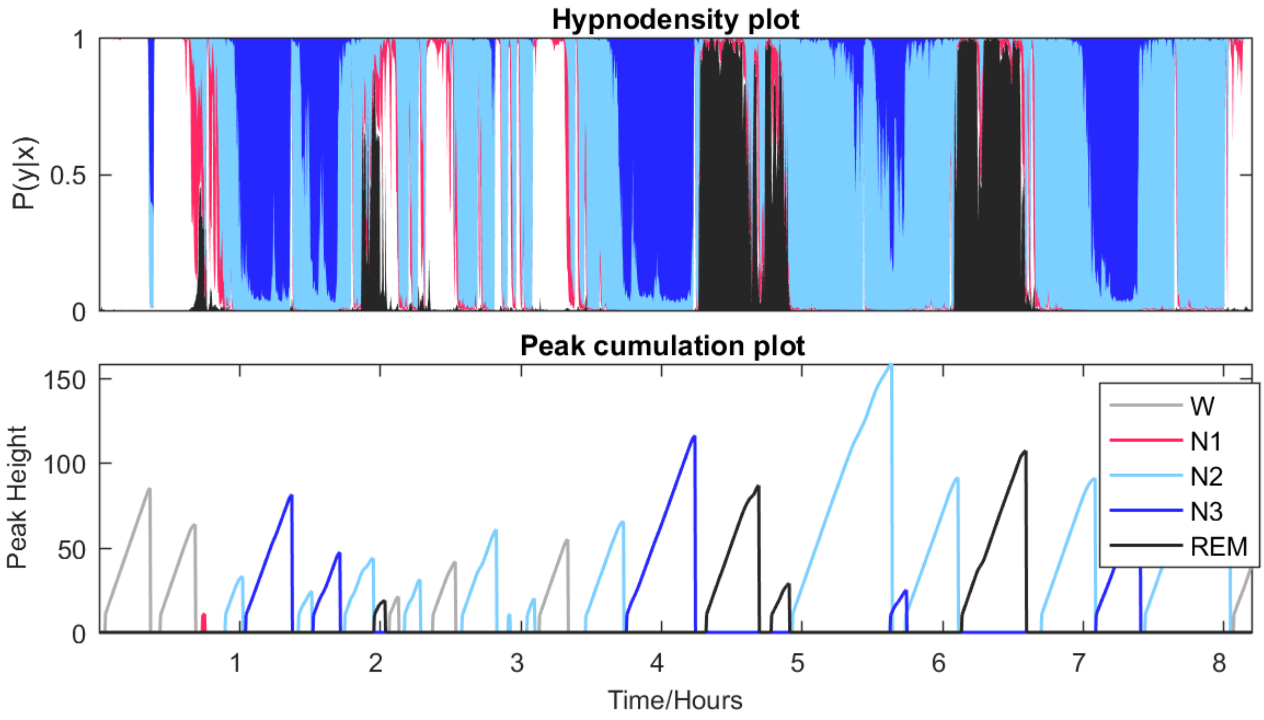

.

Supplementary Figure 4: Peak cumulation plot. It visualizes how hypnodensity-derived features are calculated (See Supplementary Table 10). Color codes: White – wake, red – N1, light blue – N2, dark blue – N3, black – REM

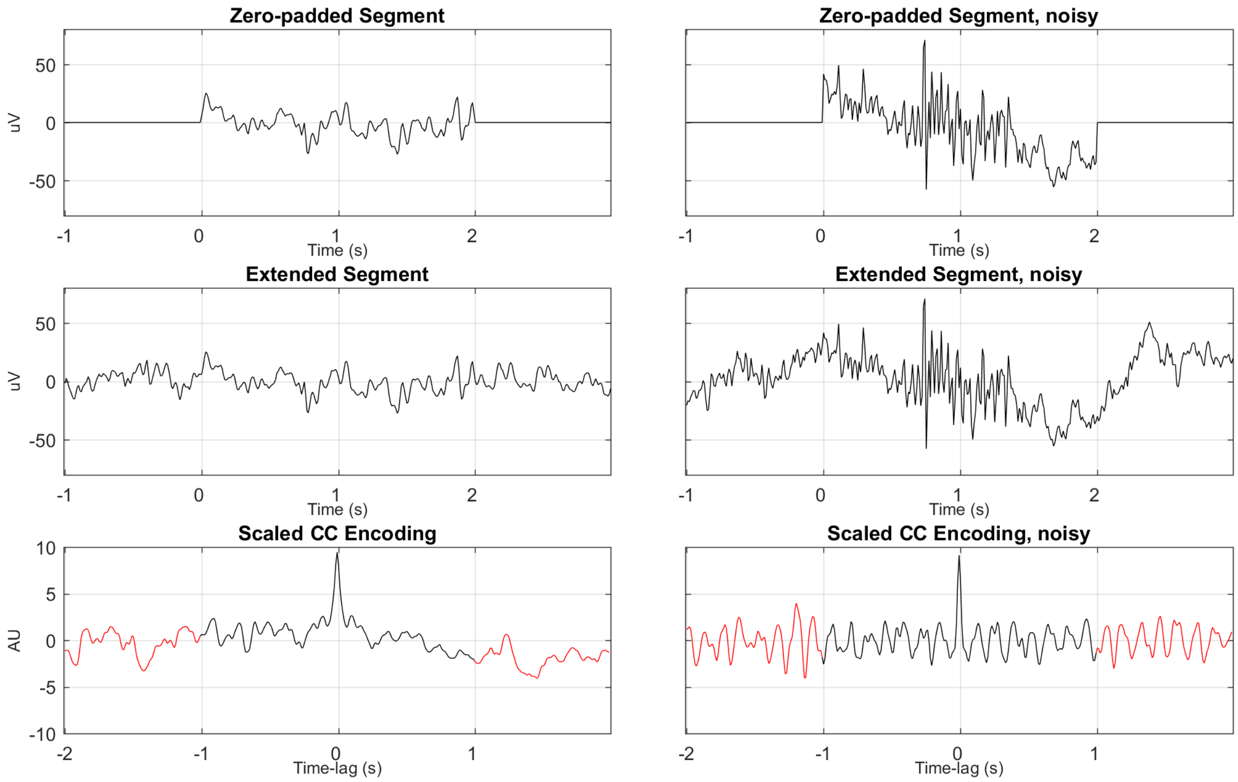

Supplementary Figure 5: Implementation of CC encoding. CC encoding of a noisy (right) and less noisy (left) signal. The central part of the encoding, representing areas of full overlap between correlated signals, is kept; the red part is discarded.

SUPPLEMENTARY TABLES

Supplementary Table 1: Description of the various cohorts included in this study and how they were used.

Cohort	Age (μ ± σ)	BMI (μ ± σ)	Sex (% male)	Sleep scoring		Narcolepsy biomarker			% narco	% hypersomnia	Use
				Train	Test	Train	Test	Replication			
**WSC**	59.7 ± 8.4	31.6 ± 7.1	53.1	1,086 (2,167 PSGs)	286	170	116	None	0.0	0.0	Training and testing of sleep scoring models and narcolepsy biomarker.
**SSC**	45.4 ± 13.8	23.9 ± 6.5	59.4	617	277	139	112	None	11.6	1.8	Training and testing of sleep scoring models and narcolepsy biomarker.
**KHC**	29.1 ± 13.2	24.1 ± 4.3	58.6	None	160	87	71	None	45.8	54.2	Sleep scoring testing, and training and testing of narcolepsy biomarker.
**AHC**	34.5 ± 13.8	25.9 ± 4.9	54.0	None	None	42 (76 PSGs)	44 (84 PSGs)	None	52.3	47.7	Training and testing of narcolepsy biomarker. 86 subjects had the first PSG recorded, and 75 had an additional second PSG. A subject was used for either training or testing.
**IS-RC**	51.1 ± 4.2	32.9 ± 9.2	0.0	None	70	None	None	None	0.0	0.0	Scored by 6 different scorers. Final assessment and validation of predictive performance for sleep scoring.
**JCTS**	53.2 ± 9.8	31.0 ± 4.4	57.1	None	None	7	None	None	100.0	0.0	Training of narcolepsy biomarker.
**IHC**	33.7 ± 17.6	-	56.7	None	None	87	61	None	47.3	50.0	Training and testing of narcolepsy biomarker.
**DHC**	33.4 ± 14.8	24.8 ± 4.9	50.0	None	None	79	None	None	26.6	48.1	Training of narcolepsy biomarker.
**FHC**	28.8 ± 15.2	24.4 ± 8.1	59.0	None	None	None	None	122	51.6	18.0	Replication of narcolepsy biomarker in never seen datasets
**CNC**	28.5 ± 16.9	23.2 ± 11.5	51.3	None	None	None	None	199	34.2	0.0	Replication of narcolepsy biomarker in never seen datasets
**Total subjects**				1,703	793	611	404	321			
**Total PSGs**				2,784	793	645	444	321			

% narco. = % of cohort with type 1 narcolepsy; % hypersomnia= % with idiopathic hypersomnia or narcolepsy type 2 (high pretest probability cohort)

Supplementary Table 2: A cumulative assessment of the scorers.

		Consensus					
	**Stages**	**Wake**	**N1**	**N2**	**N3**	**REM**	
Accumulation of Individual Scorers	Wake	13.28%<br>13.25%	1.04%<br>0.98%	0.86%<br>0.87%	0.08%<br>0.08%	0.23%<br>0.22%	0.86<br>0.86
	N1	0.79%<br>0.88%	3.36%<br>3.61%	1.23%<br>1.42%	0.03%<br>0.03%	0.29%<br>0.31%	0.59<br>0.58
	N2	0.87%<br>0.84%	2.46%<br>2.30%	44.66%<br>45.48%	4.89%<br>5.92%	0.85%<br>0.84%	0.83<br>0.82
	N3	0.05%<br>0.05%	0.02%<br>0.02%	2.58%<br>1.54%	6.45%<br>5.41%	0.002%<br>0.002%	0.71<br>0.77
	REM	0.32%<br>0.31%	1.00%<br>0.97%	1.14%<br>1.16%	0.03%<br>0.04%	13.46%<br>13.46%	0.84<br>0.84
		0.87<br>0.86	0.43<br>0.46	0.88<br>0.90	0.56<br>0.47	0.91<br>0.91	**0.81**<br>**0.81**

The top row in every cell displays the un-weighed consensus, and the bottom row displays the weighed consensus. The values in the diagonal indicate a match between scorer and consensus. The total number of scored epochs were 324,978

Supplementary Table 3: Average relative model variance, standardized to a correct wakefulness prediction, when compared to the scoring consensus. On average, the sleep classification model shows lower variance in the diagonal, which translates to a higher certainty on predicted true positives.

		Model Predictions				
	**Stages**	**Wake**	**N1**	**N2**	**N3**	**REM**
Consensus	Wake	1.00	1.16	2.25	2.12*	3.74
	N1	1.58	0.89	1.08	0.03*	1.29
	N2	3.80	1.33	0.51	0.99	1.45
	N3	0.92*	NaN*	1.36	0.58	NaN*
	REM	3.58	1.89	1.93	NaN*	1.06

*Fewer than five observations.

Supplementary Table 4: ANOVA comparing accuracy for subjects with and without various sleep disorders.

Condition	Source	Sum of squares	Degrees of freedom	p-value	Delta Mean accuracy	
Insomnia (N = 333)	Cohort	0.30	2	$3.69 \cdot 10^{-21}$		
$N_{Insomnia}$ = 134	Age	0.0026	2	0.62		
	Sex	0.0060	1	0.139	Present	0.04
	Condition	0.0003	1	0.75		
	Error	0.89	326			
OSA (N = 683)	Cohort	2.85	2	$2.81 \cdot 10^{-82}$		
N_{None} = 297	Age	0.045	2	0.020	None	-
N_{Mild} = 167	Sex	0.0018	1	0.57	Mild	0.04
$N_{Moderate}$ = 118	Condition	0.097	3	$7.53 \cdot 10^{-4}$	Moderate	0.03
N_{Severe} = 101	Error	3.82	674		Severe	0.00
RLS (N = 580)	Cohort	2.16	2	$6.50 \cdot 10^{-54}$		
N_{RLS} = 136	Age	0.056	2	0.020		
	Sex	0.011	1	0.22	Present	0.08
	Condition	0.016	1	0.13		
	Error	4.05	573			
PLMI (N = 288)	Cohort	-	-	-		
N_{None} = 120	Age	0.0027	1	0.31	None	-
N_{Mild} = 80	Sex	0.0014	1	0.45	Mild	0.00
$N_{Moderate}$ = 55	Condition	0.011	3	0.22	Moderate	-0.01
N_{Severe} = 33	Error	3.9297	282		Severe	-0.02
Narcolepsy (N = 729)	Cohort	2.05	2	$1.63 \cdot 10^{-65}$		
$N_{Narcolepsy}$ = 98	Age	0.13	2	$6.73 \cdot 10^{-6}$		
	Sex	0.018	1	0.070	Present	-0.15
	Condition	0.368	1	$1.77 \cdot 10^{-15}$		
	Error	4.01	722			
Overall (N = 729)	Cohort	2.97	2	$6.10 \cdot 10^{-82}$		
	Age	0.065	2	0.0047		
	Sex	0.010	1	0.19		
	Error	4.38	723			

The model used is the ensemble of all CC models. Each analysis is done separately to account for missing values. Cohorts are the SSC, WSC, KHC and AHC. Age is grouped as age<30, 30≤age<50 and age≥50. OSA is grouped as AHI<5, 5≤AHI<15, 15≤AHI<30 and AHI≥30. PLM is grouped as PLMI <5, 5≤ PLMI <15, 15≤ PLMI <30 and PLMI ≥30.

Supplementary Table 5: Selection frequency and descriptions of each of the 38 features included in the Gaussian process model used for narcolepsy prediction.

#	Feature # in supplementary Table 10.	Stage Combination	Relative selection frequency
**1**	12	W, N2, REM	1
**2**	Nightly SOREMPs (REM latency ≤ 15 min)		0.91
**3**	15	W	0.82
**4**	6	REM	0.82
**5**	2	W	0.68
**6**	2	N2, REM	0.68
**7**	14	W, N2	0.68
**8**	13	W, N1	0.64
**9**	5	N3	0.59
**10**	5	REM	0.59
**11**	13	N1, N2	0.59
**12**	8	N1	0.55
**13**	11	N1	0.55
**14**	7	W, N1, REM	0.55
**15**	5	W, N1, N3	0.55
**16**	6	W, N1, N3	0.55
**17**	1	W, N1, N2, REM	0.55
**18**	Hypnodensity sleep stage bout transitions: N2 to N3		0.55
**19**	Accumulation of the wakeful periods ≤ 15 minutes		0.50
**20**	Hypnodensity sleep stage bout transitions: W/N1 to REM		0.50
**21**	11	N3, REM	0.45
**22**	2	N1, REM	0.45
**23**	7	W, N2, N3	0.45
**24**	12	W	0.41
**25**	2	N1	0.41
**26**	12	N2	0.41
**27**	14	N2	0.41
**28**	7	N2, REM	0.41
**29**	8	N2, REM	0.41
**30**	6	N1, N2	0.41
**31**	15	N1, N2	0.41
**32**	15	W, N3	0.41
**33**	12	W, N1	0.41
**34**	5	W, N2, REM	0.41
**35**	1	W, N1, N3, REM	0.41
**36**	1	W, N1, N2, N3, REM	0.41
**37**	Accumulation of REM epochs following wakeful periods		0.41
**38**	Hypnodensity sleep stage bout transitions: N2 to REM		0.41

Supplementary Table 6: Descriptive statistics on the evaluation of the narcolepsy biomarker in models with and without the HLA biomarker. Performance on models with HLA typing is reported for regular threshold and optimized threshold, since the ROC curve is changed dramatically by adding HLA. Mean value and 95% confidence interval. PPV and NPV are positive and negative predictive value, respectively.

Model	Accuracy (%)	Sensitivity (%)	Specificity (%)	PPV (%)	NPV (%)	Number of PSGs	T1N fraction
**Test (T)**	0.95<br>0.92-0.97	0.91<br>0.84-0.96	0.96<br>0.93-0.98	0.88<br>0.80-0.93	0.97<br>0.95-0.99	444	0.24
**Replication (R)**	0.92<br>0.88-0.95	0.93<br>0.87-0.97	0.91<br>0.87-0.95	0.87<br>0.80-0.93	0.95<br>0.92-0.98	321	0.28
**T+R, HLA**	0.96<br>0.94-0.97	0.90<br>0.84-0.93	0.99<br>0.98-1.00	0.97<br>0.94-0.99	0.95<br>0.93-0.97	584	0.31
**T+R, HLA, optimized**	0.94<br>0.92-0.96	0.94<br>0.90-0.97	0.94<br>0.92-0.96	0.88<br>0.83-0.92	0.97<br>0.95-0.99	584	0.31
**High pre-test (HPT), no HLA.**	0.91<br>0.87-0.94	0.90<br>0.86-0.94	0.92<br>0.86-0.96	0.94<br>0.91-0.97	0.86<br>0.80-0.91	335	0.61
**HPT, HLA**	0.93<br>0.90-0.95	0.90<br>0.84-0.93	0.98<br>0.96-1.00	0.99<br>0.97-1.00	0.85<br>0.79-0.91	296	0.61
**HPT, HLA, optimized**	0.93<br>0.90-0.95	0.94<br>0.90-0.97	0.90<br>0.85-0.95	0.94<br>0.90-0.97	0.90<br>0.85-0.95	296	0.61

Supplementary Table 7: Confusion matrix on the SSC and KHC data, displaying the relationship between scorer and the ensemble estimate as a fraction of the amount of data in total.

		Target					
	**Stages**	**Wake**	**N1**	**N2**	**N3**	**REM**	
**Model prediction**	Wake	13.94%<br>8.02%<br>$2.08 \cdot 10^{-5}$	0.40%<br>0.54%<br>0.085	1.46%<br>1.59%<br>0.54	0.04%<br>0.07%<br>0.01	0.43%<br>0.59%<br>0.097	0.86<br>0.74
	N1	2.58%<br>3.59%<br>0.014	1.51%<br>1.53%<br>0.916	3.64%<br>2.70%<br>0.024	0.08%<br>0.13%<br>0.095	1.14%<br>1.57%<br>0.011	0.17<br>0.16
	N2	2.18%<br>4.07%<br>$4.59 \cdot 10^{-6}$	1.30%<br>2.79%<br>$4.86 \cdot 10^{-12}$	42.55%<br>38.59%<br>0.002	2.06%<br>1.94%<br>0.714	1.73%<br>2.18%<br>0.090	0.85<br>0.78
	N3	0.02%<br>0.02%<br>0.872	0.002%<br>0.003%<br>0.582	2.68%<br>4.05%<br>0.001	5.84%<br>7.67%<br>0.023	0.004%<br>0.009%<br>0.357	0.68<br>0.65
	REM	0.99%<br>3.03%<br>$4.07 \cdot 10^{-12}$	0.36%<br>0.71%<br>$1.43 \cdot 10^{-5}$	1.81%<br>1.91%<br>0.674	0.05%<br>0.06%<br>0.753	13.01%<br>12.64%<br>0.588	0.80<br>0.69
		0.71<br>0.43	0.42<br>0.27	0.82<br>0.79	0.72<br>0.78	0.80<br>0.74	0.77<br>0.68

The top row of each cell is data from non-narcoleptics, the second row is from narcoleptics, and the bottom row is the p-value, indicating whether there is a significant difference in the two means. 98 narcolepsy subjects and 500 non-narcolepsy subjects were used for the analysis.

Supplementary Table 8: Models tested. 32 single models are tested, and 9 ensembles, totaling 41 models.

Single models					
	**Memory**	**Seg. Len.**	**Complexity**	**Encoding**	**Realizations**
**Configuration 1**	Simple FF	5 s	Low	Octave	1
**Configuration 2**	LSTM	15 s	High	CC	2

Ensembles									
**Parameters included**	All Oct FF	All Oct LSTM	All CC FF	All CC LSTM	All FF	All LSTM	All Oct models	All CC models	All models
**N. models**	8	8	8	8	16	16	16	16	32

Supplementary Table 9: The number of stage combinations, and the number of features this leads to.

	Single stage	Two stages	Three stages	Four stages	Five stages	Additional	Total
**Combinations**	5	10	10	5	1		31
**Features**	75	150	150	75	15	16	481

Supplementary Table 10: Description of each feature, how it is calculated, and how it is numerated.

#	Description of what is expressed	Formula
**1**	General prevalence of a value	$\log\left(\frac{1}{N}\sum_{seg=1}^{N}\boldsymbol{\Phi}(\mathcal{C}_k)\right)$
**2**	Highest achieved value, measured as the distance from the highest value possible.	$-\log\left(1-\text{maximum}\left(\boldsymbol{\Phi}(\mathcal{C}_k)\right)\right)$
**3**	Measures average fluctuations in value.	$\log\left(\frac{1}{N}\sum_{seg=1}^{N}\left\lvert\frac{d\boldsymbol{\Phi}(\mathcal{C}_k)}{dseg}\right\rvert\right)$
**4**	Log of Shannon entropy, calculated through a wavelet decomposition, where s_i contains the wavelet decompositions of $\boldsymbol{\Phi}(\mathcal{C}_k)$. Measures the amount of information contained in the signal, i.e. how many different values are achieved.	$log\left(\frac{-\sum_i s_i^2\log s_i^2}{N}\right)$
**5**<br>**6**<br>**7**<br>**8**	Time until 5%, 10%, 30% or 50% of the maximum value has been achieved.	$\log\left(\text{first}_{arg>5\%,10\%,30\%,50\%}\left(\frac{\text{cumsum}(\boldsymbol{\Phi}(\mathcal{C}_k))}{\text{sum}\left(\boldsymbol{\Phi}(\mathcal{C}_k)\right)}\right)\cdot 30\right)$
**9**	Maximum value achieved weighed by the mean prevalence.	$\sqrt{\left(\text{maximum}\left(\boldsymbol{\Phi}(\mathcal{C}_k)\right)\cdot\text{mean}(\boldsymbol{\Phi}(\mathcal{C}_k))\right)}$
**10**	Average fluctuations of value weighed by mean prevalence.	$\left(\frac{1}{N}\sum_{seg=1}^{N}\left\lvert\frac{d\boldsymbol{\Phi}(\mathcal{C}_k)}{dseg}\right\rvert\right)\cdot\text{mean}(\boldsymbol{\Phi}(\mathcal{C}_k))$
**11**	Shannon entropy weighed by mean prevalence.	$log\left(\frac{-\sum_i s_i^2\log s_i^2}{N}\cdot\text{mean}(\boldsymbol{\Phi}(\mathcal{C}_k))\right)$
**12**<br>**13**<br>**14**<br>**15**	Time until 5%, 10%, 30% or 50% of the maximum value has been achieved weighed by mean prevalence.	$\sqrt{\left(\text{first}_{arg>5\%,10\%,30\%,50\%}\left(\frac{\text{cumsum}(\boldsymbol{\Phi}(\mathcal{C}_k))}{\text{sum}\left(\boldsymbol{\Phi}(\mathcal{C}_k)\right)}\right)\cdot 30\text{mean}(\boldsymbol{\Phi}(\mathcal{C}_k))\right)}$

Each individual feature is scaled by subtracting the mode dividing by the difference between the 85th and 15th percentile. Each value was assessed visually to ensure that the transformations and scaling was done optimally. cumsum is the culminative sum.
